# A genome-wide association study identified one variant associated with static spatial working memory in Chinese population

**DOI:** 10.3389/fgene.2022.915275

**Published:** 2022-09-13

**Authors:** Liming Zhang, Zijian Zhu, Qing Yang, Jingjing Zhao

**Affiliations:** School of Psychology, Shaanxi Normal University, and Shaanxi Provincial Key Research Center of Child Mental and Behavioral Health, Xi’an, China

**Keywords:** GWAS, spatial working memory, single nucleotide polymorphisms (SNPs), gene, experiment

## Abstract

Spatial working memory (SWM) is a kind of memory that temporarily preserves spatial information (the location or order of objects, etc.). Individuals with mental disorders tend to show worse performance in SWM task. This study investigated the genetic basis of two subtypes of SWM, static spatial working memory (SSWM) and dynamic spatial working memory (DSWM) in humans, using quantitative genomic analyses. A total of **451** Chinese students were tested on their magnitudes of SSWM and DSWM. A genome-wide association study (GWAS) was performed. Two SNPs (top SNP: rs80263879, *p* = 1.6 × 10^−9^, gene: *epoxide hydrolase 2*, *EPHX2*) reaching genome-wide significance for SSWM were identified. There is a high linkage disequilibrium between these two SNPs. The data of expression quantitative trait locus (eQTL) showed that different genotypes of rs80263879 and rs72478903 made significant differences in the expression of *EPHX2* gene in the spinal cord (*p* = 0.022, *p* = 0.048). Enrichment analysis identified a gene set significantly associated with DSWM. Overall, our study discovered a candidate genetic locus and gene set for the genetics of the SWM.

## Introduction

Spatial working memory (SWM) is an important component of working memory, it can produce, operate and maintain visual images, related to spatial position, motion, etc. SWM can be divided into static spatial working memory (SSWM) and dynamic spatial working memory (DSWM) (Pickering, Gathercole, Hall, & Lloyd, 2001; Cocchi et al., 2007). In the SSWM task, spatial information is usually processed once; in the DSWM task, spatial information has to be processed continuously. SWM reflects the ability to temporarily store spatial information (Goodale & Milner, 1992), varing widely across individuals. In particular, individuals with mental disorders, such as autism spectrum disorder (ASD) or schizophrenia, have varying degrees of impairment in SWM (Jiang et al., 2015; Minor and Park, 1999). The reason may be that the development process of SWM in patients with mental diseases from their 10th to 20th years of age has stagnated (Song et al., 2013).

What are the factors causing the individual differences of SWM ability? First, there is a research evidence that environmental factors (educational level, age, etc.) influence SWM ability, these environmental factors were significantly correlated with SWM ability in healthy subjects, but not in schizophrenic subjects (Stratta et al., 2001). Second, studies revealed that genetic factors influenced SWM ability in schizophrenic patients. For instance, researchers divided schizophrenic patients into different categories according to different genotypes of a specific gene (catechol-O-methyltransferase) (Miskowiak et al., 2017). Different groups of subjects performed differently in SWM task. However, so far, there is no genetic research on SWM in the general population and at the genome-wide level.

In this study, we did a GWAS among high school students and college students to reveal the molecular mechanism of SWM. We analyzed two SWM subtypes (i.e., SSWM and DSWM). We investigated the function of loci significantly associated with the phenotype. We also performed enrichment analysis to see if phenotypes were affected by the joint effects of multiple loci or genes. Our results showed that SWM has a specific genetic mechanism.

## Methods

### Participants

Cohorts consisted of college students recruited from the Shaanxi Normal University and Xi’an Jiaotong University in Northwest of China, and senior high school students recruited from Sichuan LuXian No.2 High School in Southwest of China and Xi'an No. 1 middle school in Northwest of China. There were 544 participants in total, with 100% of Han population. The average age is 17 years old (standard deviation, SD = 1.32), with 54% of female. Each participant had no history of mental illness, and none of the subjects reported psychiatric or visual illness. All subjects had normal visual acuity or corrected visual acuity.

The experimental procedure was approved by the evaluation committee of Shaanxi Normal University. The subjects were clear about the purpose of the experiment. They signed informed consent before the experiment.

### Behavioral assay

**FIGURE 1:**
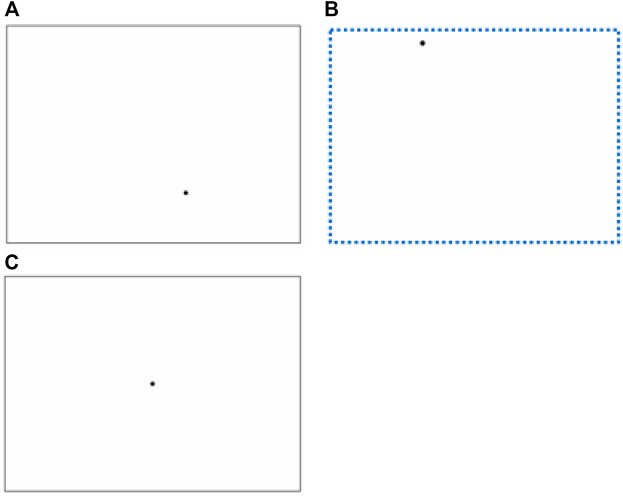
Stimuli in experiment. The above three figures reveal ‘Stimuli in the learning stage of SSWM task’ **(A)**, ‘Stimuli in the learning stage of DSWM task’ **(B)** (There are no dashed tracks in the formal experiment), ‘Stimuli in the test stage of SSWM and DSWM task’ **(C)**.

***Stimuli.*** Three stimuli of SWM were created, Figures 1A,C for the SSWM task, Figures 1B,C for the DSWM task. Each SWM stimulus was composed of a rectangular box and a dot, and was presented on a white backgroud. Figures 1A,B are the learning stage stimuli, Figure 1C is the test stage stimuli.

The stimuli were generated and presented by MATLAB and its toolbox (PsychToolbox). The size of the LCD screen of the stimulating computer is 23.8 inches and the refresh rate is 60 Hz. The viewing distance was kept at 40 cm using a chin rest. Luminance for all participants remained unchanged.

***Procedure.*** SSWM task: The experiment is based on Gordon’s spatial positioning test (Gordon, 1986). Specific procedure was as follows. At first, a rectangular box with a certain size was randomly presented at different positions on the computer screen, and at the same time, a dot with a certain size was randomly presented at different position of the rectangular. After a few seconds, these stimulus disappeared automatically, as shown in Figure 1A; then a rectangular box and a dot at its center with the same size appeared on the screen, as shown in the Figure 1C. The participants’ task was to move the dot in the center of the rectangular box to the position in the rectangular box where the dot just disappeared through the direction key on the keyboard. The position of the rectangular box on the screen is different in the learning stage and the testing stage, so as to avoid the subjects completing the task by remembering the coordinate position of the point in the whole screen.

The specific operation of DSWM task is that a dot randomly appeared in the upper left area of the screen and completed the following movements in sequence at a fixed speed: a centimeters from left to right → b centimeters from top to bottom → a centimeters from right to left → b centimeters from bottom to top. This dot moved to its original place and then disappeared. The motion trajectory of dot finally formed a rectangle box with the same size as that in the SSWM task. After the dot disappeared, another dot appeared in the disappeared motion track (Figure 1B). After the latter dot disappeared, participants completed the same task as the SSWM experiment.

This task has a total of 50 attempts and takes a total of 40 min. The measurement index of this task is the distance deviation between the position of the last dot and the position of the initial dot, that is, how many screen pixels are apart.

### Genotype quality control and imputation

DNA was extracted from saliva samples of 520 participants, and individuals were genotyped using Illumina Asian screening array (650K) by Beijing Compass Biotechnology (N1 = 288) and Genergy Biotechnology (N2 = 232). The quality control of two samples from different companies is exactly the same. Briefly, SNPs were filtered out if they showed a variant call rate <0.9, a minor allele frequency (MAF) < 0.05, a hardy-weinberg equilibrium (HWE) < 10^−5^. Individuals were filtered out if they showed a missing genotype data (mind) < **0.10** (3 people from sample1 were removed, 1 people from sample2 were removed), unexpected duplicates or probable relatives (PI-HAT>0.20) (Anderson et al., 2010; Chang et al., 2015).

For imputation, autosomal variants were aligned to the 1000G genomes phase 1v3 reference panel. Imputation was performed using Michigan imputation Server 4.0 in 5 Mb chunks with 500 kb buffers (Das et al., 2016), filtering out variants that were monomorphic in the Genome Asia Pilot (GAsP). Chunks with 51% genotyped variants or concordance rate <0.92 were fused with neighboring chunks and re-imputed. Imputed variants were filtered out for rsq <0.60, MAF<0.05, mind<0.1, HWE<10^−5^ using Plink (v1.90).

After imputation, we merged two gene samples, and the merged sample left 516 individuals and 4 196 499 SNPs.

### Genome-wide association analyses

After completing the above operations, **451** individuals have genetic data, phenotypic data and covariate data at the same time. Genome-wide association analyses were performed using Plink 1.90 (Rentería et al., 2013), fitting an additive model to the linear regression model with adjustment for sex, grade, and the first two principle components of population structure (Chang et al., 2015). Manhattan plots and Quantile-quantile plots were generated using the ggplot2 package in R. We used locuszoom to generate regional association plots (Pruim et al., 2010). The sign of genome-wide significance is *p* < 5 × 10^−8^.

### Bioinformatics analysis

In order to examine whether the discovered rs80263879 and rs72478903 influnce gene expression, we checked the expression quantitative trait locus (eQTL) for these two loci in the Genotype-Tissue Expression (GTEx) Cohort (https://www.gtexportal.org/home/) (GTEx Consortium, 2020). Analysis of eQTL can be with reference to the study of Ramasamy et al. (Ramasamy et al., 2014).

### Gene- and pathway-based enrichment tests

Gene-based and pathway-based enrichment tests for SSWM and DSWM were conducted by MAGMA (de Leeuw et al., 2015). Gene-based analyses is based on GWAS data. Every SNP is located on the protein-coding gene according to NCBI37.3. Genes were analyzed after internal quality control. We adopted default internal quality control steps and internal quality control values of magma. A total of 17,225 genes were involved in the analysis, so the threshold of significance was set at 2.90 × 10^−6^ (*p* = 0.05/17,225).

Pathway-based analyses are derived from the results of gene-based analyses using a competitive gene-set analysis (Gerring et al., 2019). Orginal pathway were from Molecular Signatures Database website (MSigDB, c2.all.v7.0.entrez). A total of 5 497 pathways were involved in the analysis, so the threshold of significance was set at 9.10 × 10^−6^ (*p* = 0.05/5 497).

## Results

### Characteristics of behavioral data

**FIGURE 2:**
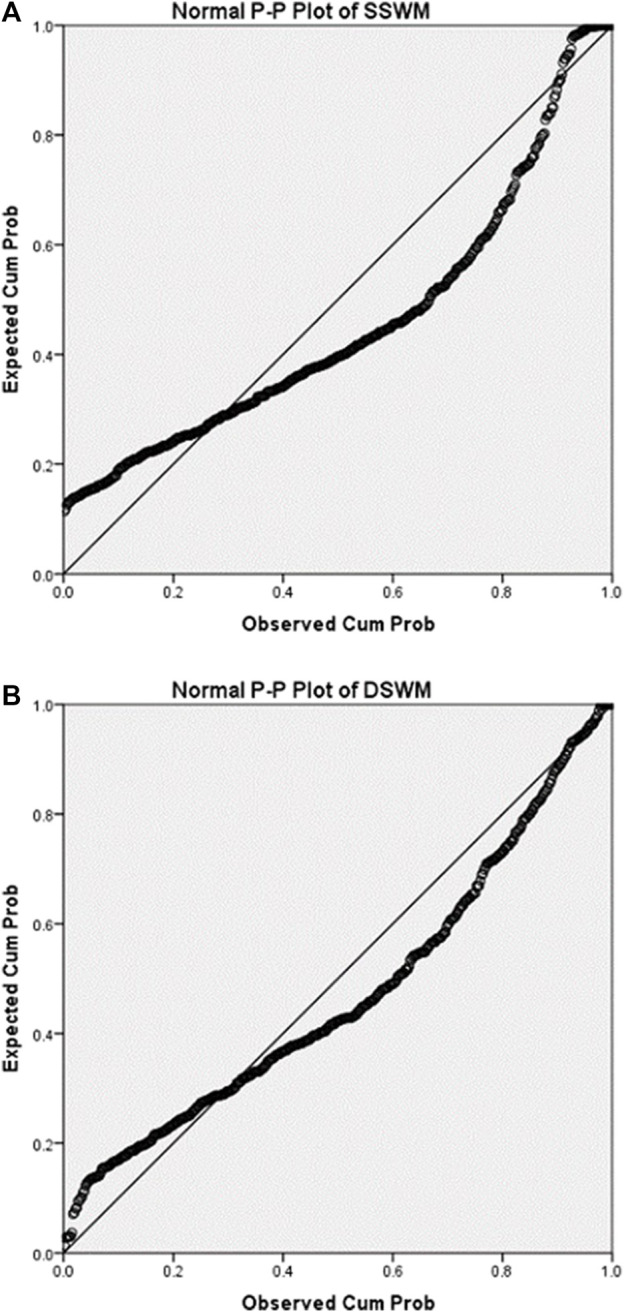
P-P plots for phenotypes. The above two figures summarise ‘consistency between the cumulative proportion of data of DSWM and the cumulative proportion of normal distribution’ **(A)**, ‘consistency between the cumulative proportion of data of SSWM and the cumulative proportion of normal distribution’ **(B)**. The closer the scatter distribution is to the diagonal, the more the data conforms to the normal distribution.

After phenotypic data were collected, we used P-P plot to draw the consistency of the cummulative proportion of SSWM (Figure 2A) and DSWM (Figure 2B) with the cumulative proportion of normal distribution. P-P plot shows that the distributions of the two phenotypes are a little skewed. Then, we did correlation analysis between phenotypes and sociodemographic variables (gender and grade) (Table 1). Results showed that there were significant correlations between SSWM task and sociodemographic variables.

**TABLE 1:** Correlations for sociodemographic variables (gender and grade) and phenotypes (SSWM and DSWM).

	SSWM	DSWM
Gender	-0.103^*^(a)	-0.015(a)
Grade	-0.432^**^(b)	-0.067(b)

* means *p* < 0.05, ** means *p* < 0.01, a means Pearson’s r, b means Spearman’s rho.

### Genome-wide study of single-marker association

We identified two genome-wide significant loci associated with SSWM (rs80263879, *p* = 1.6 × 10^−9^; rs72478903, *p* = 3.5 × 10^−8^). These two loci have strong linkage disequilibrium. And these two SNPs are all located within the gene *EPHX2*. We have not identified any SNP significantly associated with DSWM. However, rs80263879 revealed weak association with DSWM (*p* = 4.5 × 10^−2^).

**FIGURE 3:**
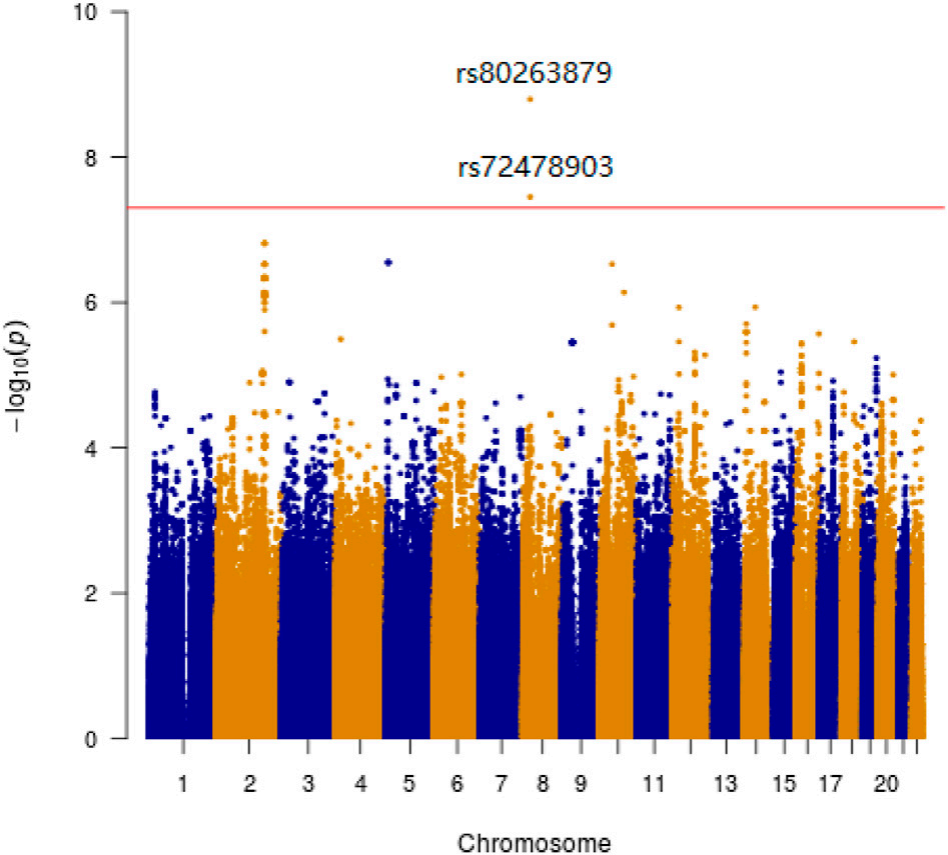
Manhattan plot showing genome-wide association analysis for SSWM. X-axis represents the position of SNPs on the chromosome, y-axis represents the significance level of association analysis. The horizontal red line represents the genome-wide significance level threshold (5 × 10^−8^).

**FIGURE 4:**
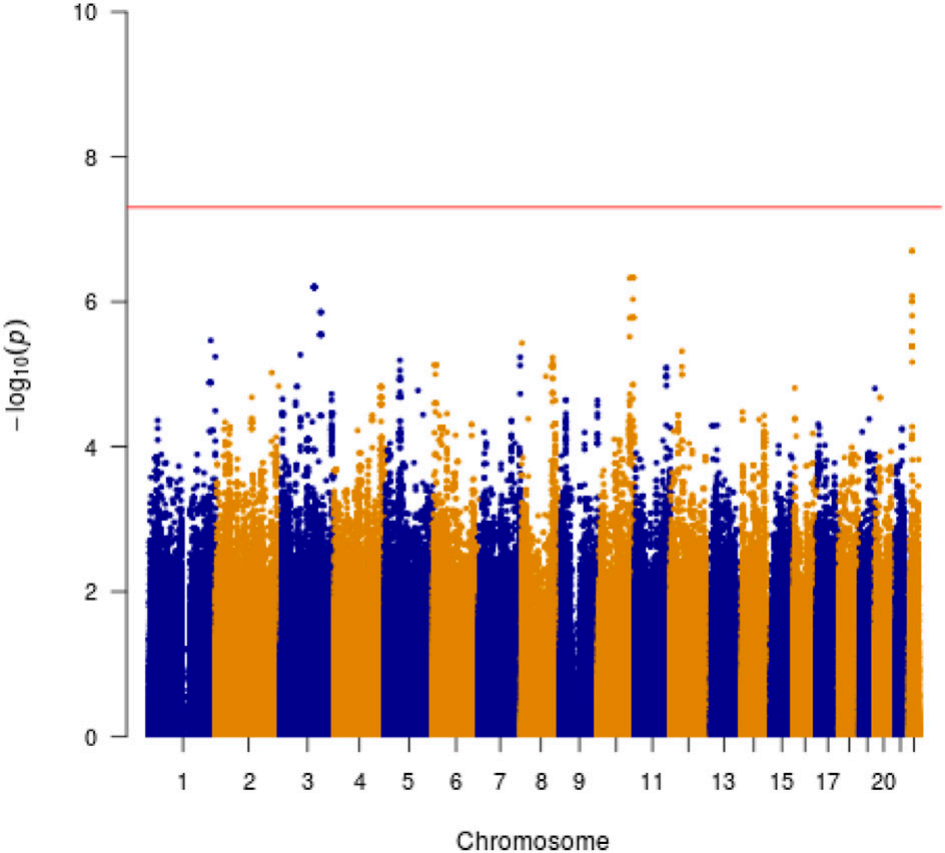
Manhattan plot showing genome-wide association analysis for DSWM.

**FIGURE 5:**
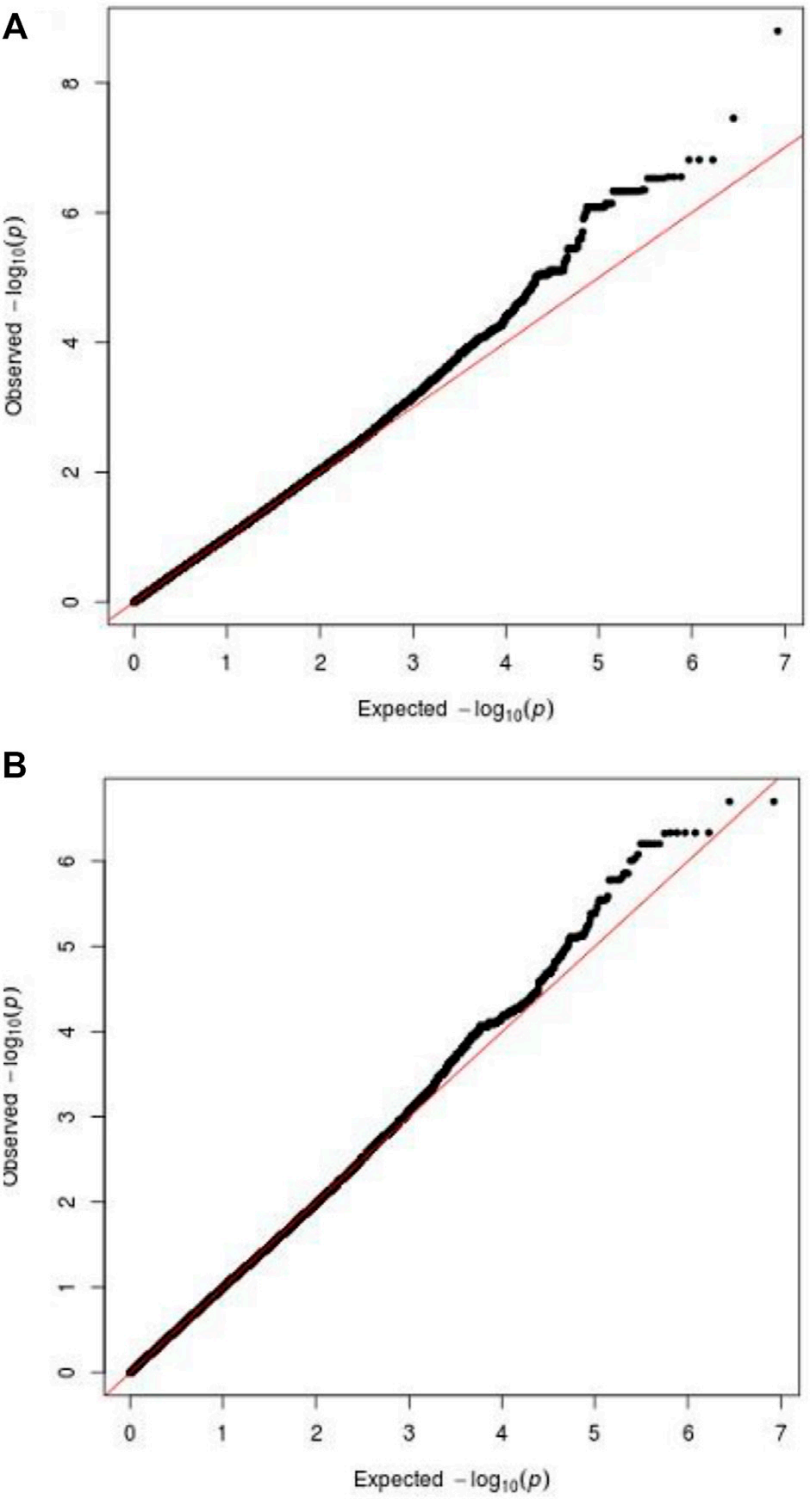
Q-Q plot for phenotypes. The above two figures show ‘Q-Q plot for SSWM’ **(A)**, ‘Q-Q plot for DSWM’ **(B)**.

**FIGURE 6:**
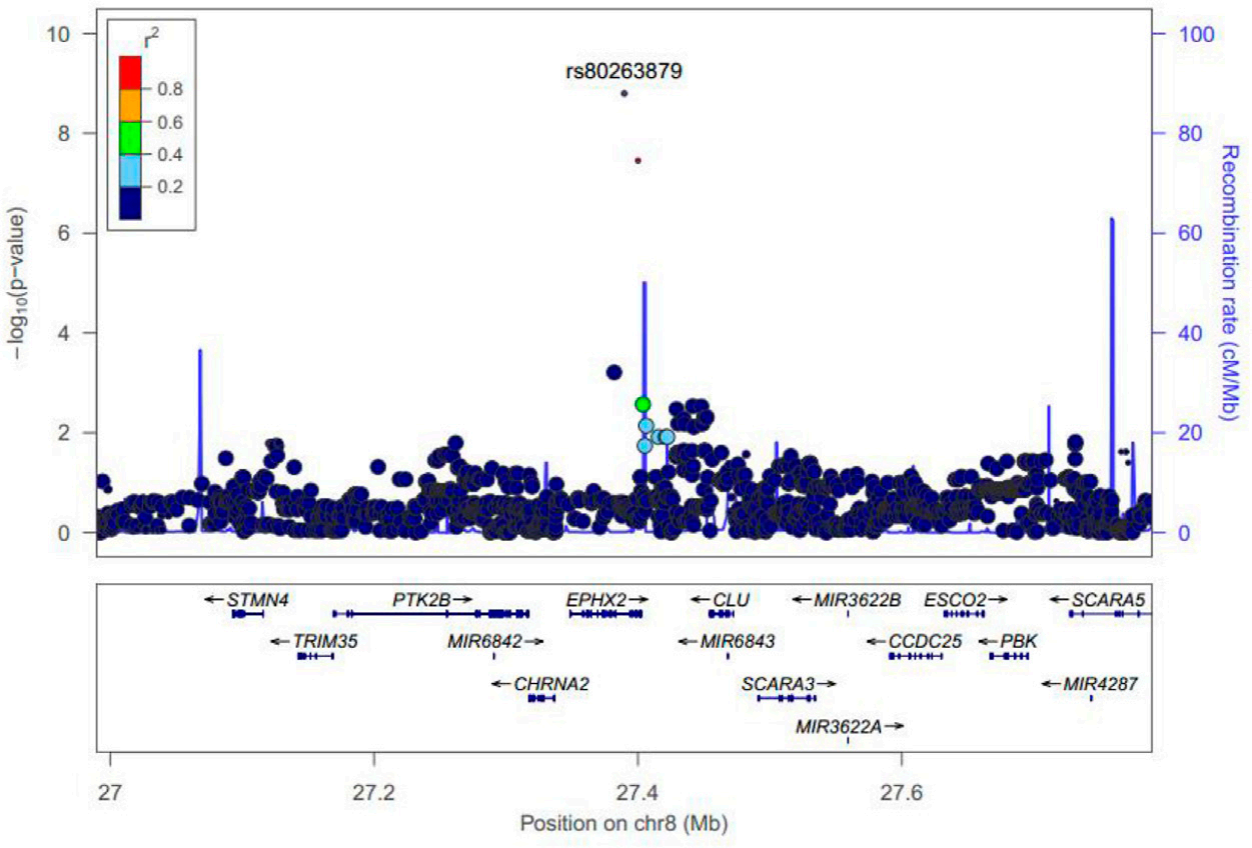
Regional plot of rs80263879 for SSWM. The most significant locus was marked violet. *r* ^2^ is the LD value of rs80263879 and any other locus.

Manhattan plots were adopted to reveal the results of association analysis for SSWM (Figure 3) and DSWM (Figure 4). Due to the quality control of the two samples, the specific number of people at each locus is somewhat different. More details for significant locus were reported in Table 2 **.** We used quantile-quantile plots for SSWM (Figure 5A) and DSWM (Figure 5B) to reveal the rationality of their respective analysis models. The genomic inflation factors λ of quantile-quantile plots was 1.023 for SSWM and 1.013 for DSWM, confirming little influence of population stratification. Regional association plot of the significant locus are revealed in Figure 6.

**TABLE 2:** Two lead single-variant associations detected in the GWAS analyses.

SNP	CHR:BP	ALLEL1	Test	NMISS	BETA	STAT	P
rs80263879	8:27389631	A	ADD	188	19.73	6.358	1.6 × 10^−9^
rs72478903	8:27400068	A	ADD	188	17.82	5.759	3.5 × 10^−8^

### Genetic mechanism of rs80263879 and rs72478903

**FIGURE 7:**
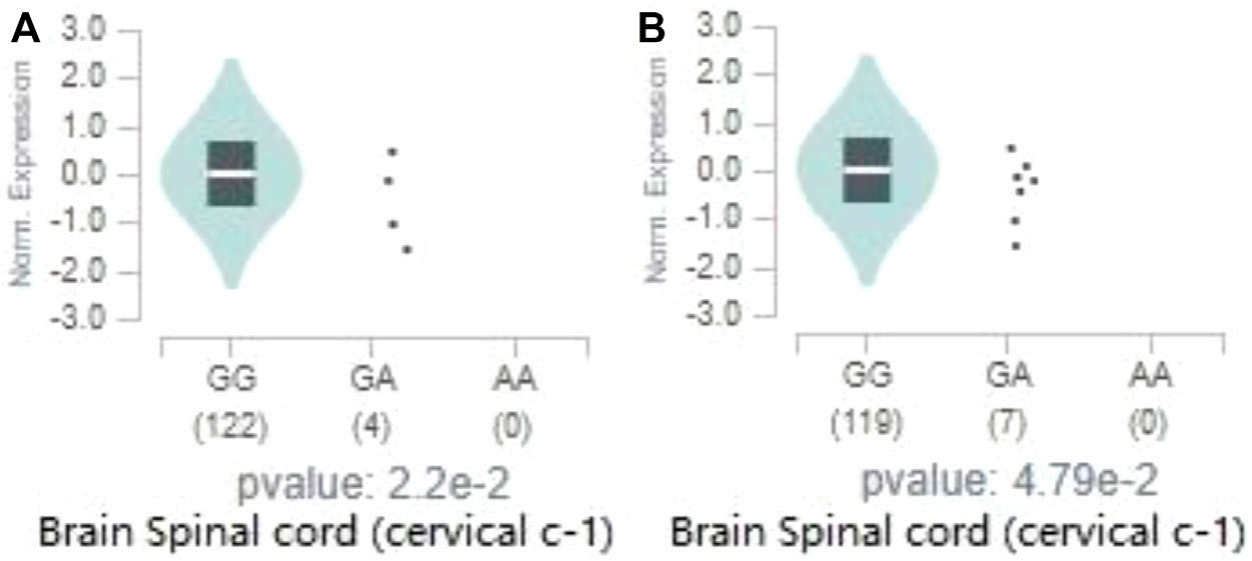
Effect of significant loci on the expression of *EPHX2*. The above two figures reveal ‘expression amount of *EPHX2* on different genotypes of rs80263879 in spinal cord (cervical c-1)’**(A)**, ‘expression amount of *EPHX2* on different genotypes of rs72478903 in spinal cord (cervical c-1)’**(B)** from Genotype-Tissue Expression (GTEx) Cohort.

Two genome-wide significant loci we identified are located in gene *EPHX2*. According to the data of eQTL from GTEx, both loci have a significant regulatory effect on the expression of *EPHX2* in the spinal cord (cervical c-1) (rs80263879, *p* = 2.2 × 10^−2^; rs72478903, *p* = 4.8 × 10^−2^). Figure 7A reveals that the major allele G of rs80263879 is associated with increased *EPHX2* expression in spinal cord (cervical c-1). Results of eQTL in other 12 brain regions of rs80263879 are revealed in Supplementary Figure S1. Similarly, Figure 7B reveals that the major allele G of rs72478903 is associated with increased *EPHX2* expression in **spinal cord (cervical c-1).** Results of eQTL in other 12 brain regions of rs72478903 are revealed in Supplementary Figure S2.

### Gene- and pathway-based associations

**FIGURE 8:**
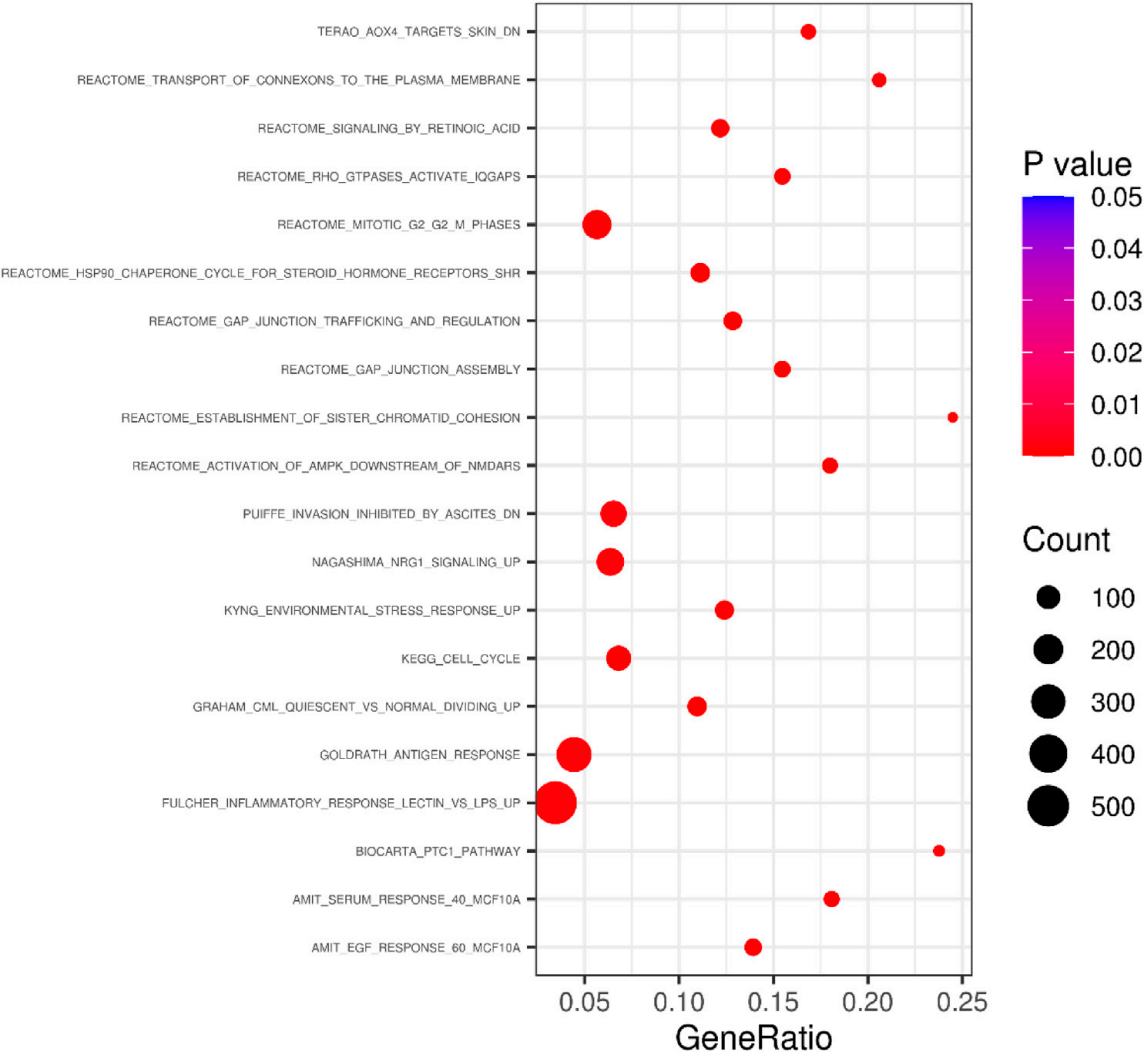
Bubble plot summarising the results of the pathway-based enrichment tests for DSWM. The plot reveals 20 pathways with the lowest *p* values.

In the pathway-based enrichment analysis, we found a pathway (Standard name: GRAHAM_CML_QUIESCENT_VS._NORMAL_DIVIDING_UP) significantly enriched with DSWM after Bonferroni correction (β = 0.51, se = 0.11, *p* = 1.36 × 10^−6^ < 9.10×10^−6^). Genes on the pathway in our data are shown in Supplementary Table S1. Genes of the pathway were found to be up-regulated in certain leukemia-derived quescent hemopoietic stem cells expressing CD34 in contrast with dividing cells supplied by the normal population (Graham, Vass, Holyoake, & Graham, 2010). Bubble plot of the 20 pathways with the lowest *p* values was shown in Figure 8, and full results for pathway-based enrichment analysis were shown in Supplementary Table S2 **.**

## Discussion

This study is the first genome-wide association study on spatial working memory in the general population. We reported two genome-wide significant loci (rs80263879 and rs72478903) associated with static spatial working memory. These two loci have strong linkage disequilibrium. We also found that rs80263879 and rs72478903 affect the expression of *EPHX2* in the spine. In addition, we identified a gene set significantly associated with dynamic spatial working memory. These results provide evidence for the genetic basis of spatial working memory.

Spatial working memory is an important ability in people's daily life, in the field of aerospace for predicting flight performance (Tirre & Raoufl 1998), and in representing an endophenotype of mental disorders such as schizophrenia. Spatial working memory has also been suggested to be influenced by genetic factors. Indeed, researchers have conducted genome-wide association analysis in schizophrenic patients and controls, and identified some loci associated with spatial working memory (Ren et al., 2015). However, so far, no genome-wide association analysis of spatial working memory has been conducted in general population. Here, our study report the first-hand evidence for the genetic basis of spatial working memory in a population of Chinese young adults.

We identified two genome-wide significant SNPs with strong LD among each other associated with static spatial working memory. These two loci are located within the gene *EPHX2*. *EPHX2* is associated with familial hypercholesterolemia (Pillai, Shah, Reddy, Ashavaid, & Vishwanathan, 2022). This was the first time that it had been found to be related to spatial working memory. Spatial working memory is usually impaired among mental disorders such as schizophrenia and ASD (Jiang et al., 2015). Expression of *EPHX2* mRNA from schizophrenia and ASD have been found higher than that of controls (Ma et al., 2019). Therefore, we speculate that EPHX2 may play its role in mental disorders through spatial working memory. However, this speculation requires further empirical evidence for validation.

Different genotypes of rs80263879 and rs72478903 make gene *EPHX2* express in different degrees in the spinal cord, indicating that the spinal cord may be associated with SWM. Research on mice revealed that spinal cord injury can significantly block the expression of dopamine receptors in the frontal lobe, thus damaging SWM (Kheyrkhah et al., 2020). However, effects of these loci on regulating the expression of the gene *EPHX2* are weak, and number of subjects with “GA” genotype is small. Therefore, more samples are needed to verify this regulatory effect in the future.

A gene set significantly associated with dynamic spatial working memory was identified in enrichment analysis. The expression of genes in this gene set is positively regulated in quiescent CD34 + cells from some leukemia. CD34 + cells can differentiate into endothelial progenitor cells, which can be conducive to the repair of patients’ myocardium (Kim et al., 2016).

In conclusion, we identified two loci significantly associated with static spatial working memory, which are located on the gene *EPHX2* and regulate the expression of this gene. We also identified a gene set associated with dynamic spatial working memory. Our research deepens the understanding of the genetic basis of spatial working memory and can provide reference for the treatment of mental diseases to some extent.

## Data Availability

The original contributions presented in the study are publicly available. This data can be found in the GWA catalog http://ftp.ebi.ac.uk/pub/databases/gwas/summary_statistics/ GCST90133001-GCST90134000, GCST90133162, and GCST90133163.
