# Supplementary figures and images for: A genome-wide association study identified one variant associated with static spatial working memory in Chinese population
Source: Front Genet. 2022 Sep 13;13:915275. doi: 10.3389/fgene.2022.915275 (PMC9514234; doi:10.3389/fgene.2022.915275)

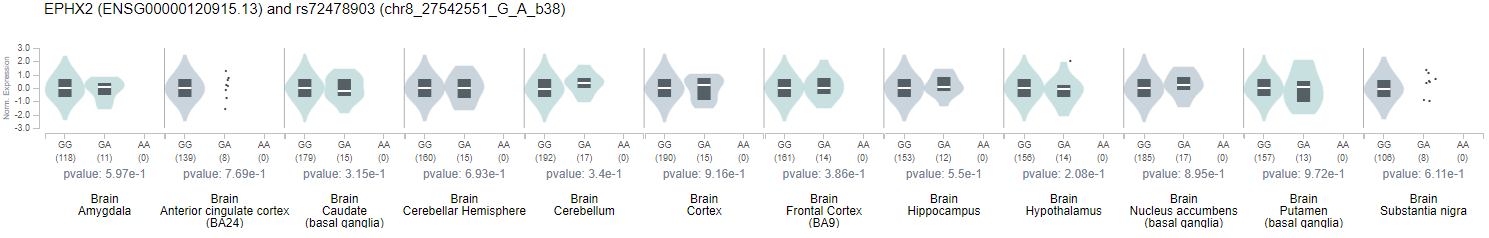

Supplement: Supplementary file 2 [file Image2.PNG]

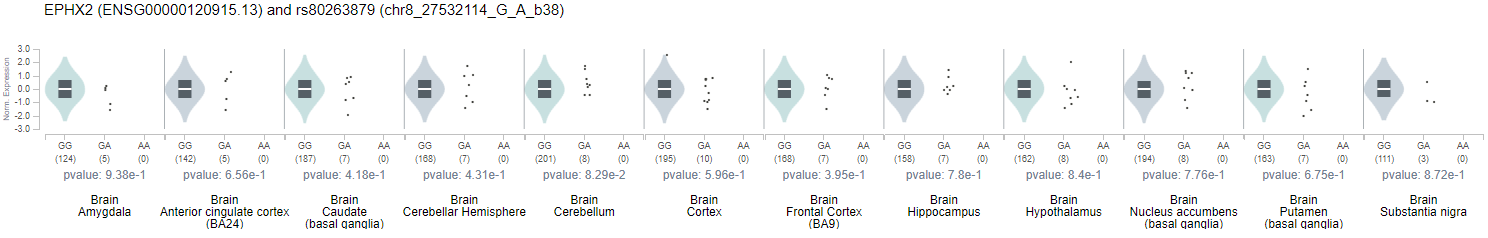

Supplement: Supplementary file 4 [file Image1.PNG]
